# Dominance and the potential for seasonally balanced polymorphism

**DOI:** 10.1101/2023.11.20.567918

**Published:** 2023-11-21

**Authors:** Evgeny Brud

**Affiliations:** Department of Biological Sciences, North Carolina State University, Raleigh, NC, USA

## Abstract

Genic dominance is a key component of fitness in diploid genotypes. Modelers exploring the conditions for balanced polymorphism under seasonal selection have argued that a reversal of dominance (where the fitness regime cyclically alternates the direction of dominance between a pair of alleles) is a powerful stabilizer of biallelic variation across a broad space of selection intensities. An alternative genetic mechanism, cumulative overdominance (in which the fitness regime maintains a constant direction of dominance), has been argued to preferentially stabilize alleles characterized by strong selection intensities, while requiring an implausibly strict parity under weak selection. Previous analytical conclusions were typically made under the assumption of symmetries for the dominance parameters. Here I investigate generalized dominance schemes for a bivoltine population in order to compare the proportional contribution of these genetic mechanisms to the stabilization of selective polymorphism. In particular, I derive the potential for polymorphism (a measure of the total parameter space conferring stability) for the generalized sex-independent model in four parameters.

## Introduction

Interest in cyclic regimes of natural selection has renewed in recent years, especially with the discovery of hundreds to thousands of allelic oscillations in temperate Drosophila melanogaster, where biallelic sites spread across the genome exhibited seasonal changes of allele frequency of ~5–20% (Bergland et al. 2014, Machado et al. 2021). Results from the deterministic theory of cyclical selection in diploid bivoltine and multivoltine organisms (2 or 3+ generations per year, respectively) include the conditions for protected polymorphism at a single-locus (sensu Prout 1968), with models assuming fitnesses to be either sex-independent (Haldane and Jayakar 1963, Bertram and Masel 2019, Ewing 1977, 1979, Hoekstra 1975, Nagylaki 1975, Dempster 1955, Gillespie and Langley 1974, Frank and Slatkin 1990) or sex-dependent (Reinhold 1999,^2000^, Yamamichi and Hoso 2017); see Johnson et al. (2023) for a recent review of theory and data on fluctuating selection. Notably, Haldane and Jayakar (1963) derived the conditions for balanced polymorphism in the general sex-independent model: the geometric mean fitnesses for each of the homozygous genotypes must be less than that of the heterozygote if polymorphism is to establish in the population. More recent work on the topic focuses on the role of season-specific dominance (a component of fitness, along with season-specific selection coefficients) in stabilizing variation (Bertram and Masel 2019; see Wittmann et al. 2017 for multilocus results).

Bertram and Masel (2019) examined the potential for biallelic polymorphism owing to two genetic mechanisms (reversal of dominance, cumulative overdominance) and two ecological mechanisms (boom-bust demography, non-overlapping generations). In their examination of the genetic mechanisms, they derived the conditions for protected polymorphism and compared the sizes of the stability-yielding parameter regions when plotted for particular values of the dominance coefficient (h_1_) in season 1 and the dominance coefficient (h_2_) in season 2 (Table 1). Four parameters being present, they simplified matters by assuming either: (i) equality of dominance parameters (h_1_=h_2_, which corresponds to symmetric reversal of dominance) or (ii) that the season 1 dominance is the complement of the season 2 dominance (${\text{h}}_{1}=1-{\text{h}}_{2}$, cumulative overdominance; Dempster 1955). The pattern that emerges from plotting given dominance values under these assumptions is that (1) the stability region for cumulative overdominance tends to the line s_1_ = s_2_ as the selection coefficients become small, and (2) dominance reversal, at least for h_1_, h_2_ less than and not too close to one-half, is far more permissive of unequal selection coefficients, with broad intervals of s_1_, s_2_ even for weak selection. Indeed, for complete beneficial dominance reversal (h_1_ =h=_2_ =0), any combination of selection coefficients yields stable polymorphism. The authors conclude that cumulative overdominance preferentially stabilizes large-effect alleles, and hence may be responsible for the Bergland et al. (2014) result of many large oscillations. While dominance reversal also stabilizes large-effect alleles, the permissive inclusion of small-effect alleles would result in a genetic architecture of seasonal fitness (assuming diminishing returns epistasis, Wittmann et al. 2017) that is composed predominantly of small oscillations. Dominance reversal thus suffers from a “weak allele problem”, they conclude, and therefore is an implausible mechanism for the strong genome-wide seasonal evolution of allele frequencies. Superior explanations incorporate a mechanism for the preferential exclusion of small-effect alleles, as occurs for cumulative overdominance and the ecological mechanisms.

Putting aside the multilocus and ecological aspects, here I focus on a broader comparison of the two genetic mechanisms in the single-locus model to see if the general pattern just described holds for violations of the simplifying assumptions (i) and (ii) above, or in other words, for generalized dominance. I ask: what is the proportional contribution of each class of dominance scheme (reversal of dominance, cumulative overdominance) to the establishment of seasonally balanced polymorphism?

For the generalized sex-independent model in four parameters (s_1_, h_1_, s_2_, h_2_), I show that a complete treatment for weak selection is possible based on geometric reasoning about the size of parameter space conferring stability, this size being the ‘potential for polymorphism’ (Asmussen and Basnayake 1990, Trotter and Spencer 2007).

## Model and Results

Consider a bivoltine population with discrete, non-overlapping generations that is subject to a two-season selection regime. Without loss of generality, suppose that the season 1 selection coefficient is greater than or equal to that of season 2. Following Haldane and Jayakar (1963), the conditions for a protected biallelic polymorphism are given by the annual fitnesses conforming to the inequality W(Aa) > W(AA), W(aa), or in terms of the parameters {W(AA, spring) = 1, W(AA, fall) = 1 − s_2_, W(Aa, spring)=1 − h_1_s_1_, W(Aa, fall) =1 − h_2_s_2_, W(aa, spring) = 1 − s_1_, W(aa, fall) = 1}:

(1) $$\left(1-{\text{h}}_{1}{\text{ s}}_{1}\right)\left(1-{\text{h}}_{2}{\text{ s}}_{2}\right)>\left(1-{\text{s}}_{2}\right)\geq \left(1-{\text{s}}_{1}\right)$$

This reduces to the Stable Polymorphism (SP) conditions:

(2) $$0<{\text{s}}_{1}\leq 1\wedge 0<{\text{s}}_{2}\leq {\text{s}}_{1}\wedge 0\leq {\text{h}}_{2}<1\wedge 0\leq {\text{h}}_{1}<\frac{{\text{s}}_{2}\left(1-{\text{h}}_{2}\right)}{{\text{s}}_{1}\left(1-{\text{h}}_{2}{\text{ s}}_{2}\right)}$$

**Figure 1.**
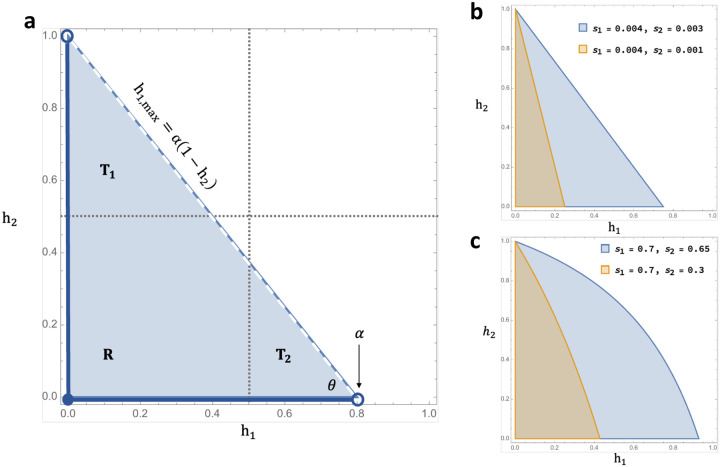
Stability regions of the dominance unit square. **(a)** Letting the season 1 selection coefficient equal 0.5% and the season 2 coefficient equal 0.4%, the stable region intersects the unit square to form a right triangle **T** (shaded region); open circles and the blue dashed line are excluded from the stable region. The quadrants of the unit square divide **T** into 3 regions **(T**_**1**_**, T**_**2**_**, R). (b)** The stability region narrows for increasing asymmetry between the s_1_, s_2_ parameters, as seen in reduction of area for α = 0.25 as compared to α = 0.75. **(c)** Extreme selection parameters (especially as α → 1) induce curvature in the boundary of the stability regions.

Let α equal the ratio of selection coefficients $\frac{{\text{s}}_{2}}{{\text{s}}_{1}}$. These inequalities imply the following:

Lemma 1: For weak selection coefficients, the SP conditions cover the unit square of dominance parameters in the form of a right triangle **T** with vertices at (0, 0),(0, 1), and (α, 0). (Figure 1).

Proof:

(0, 0) is a vertex since the SP conditions include the origin (h_1_ = h_2_ = 0). (0, 1) is a vertex since the permissible h_2_ parameter interval is [0,1). (α, 0) is a vertex since the SP conditions cross the h_1_ axis when h_2_ = 0 at:

(3) $${\frac{{\text{s}}_{2}\left(1-{\text{h}}_{2}\right)}{{\text{s}}_{1}\left(1-{\text{h}}_{2}{\text{ s}}_{2}\right)}|}_{{\text{h}}_{2}=0}=\alpha$$

The vertices (α, 0) and (0, 1) are connected by a line if we ignore products of selection coefficients. That is, the upper bound of permissible h_1_ (h_1_, _max_) conforms to:

(4) $${\text{h}}_{1,\text{max}}=\frac{{\text{s}}_{2}\left(1-{\text{h}}_{2}\right)}{{\text{s}}_{1}\left(1-{\text{h}}_{2}{\text{ s}}_{2}\right)}\approx \frac{{\text{s}}_{2}\left(1-{\text{h}}_{2}\right)}{{\text{s}}_{1}}=\alpha \left(1-{\text{h}}_{2}\right)$$

Corollary 1: For weak selection coefficients, **T** does not intersect the upper right quadrant of the unit square for which $\frac{1}{2}<{\text{h}}_{1}\leq 1\wedge \frac{1}{2}<{\text{h}}_{2}\leq 1$ (the region associated with *deleterious* reversal of dominance).

Proof: Ignoring second-order terms, ${\text{h}}_{1}<\frac{{\text{s}}_{2}\left(1-{\text{h}}_{2}\right)}{{\text{s}}_{1}\left(1-{\text{h}}_{2}{\text{ s}}_{2}\right)}$ reduces to ${\text{h}}_{1}<\alpha \left(1-{\text{h}}_{2}\right)$. By assumption α ≤ 1, and so ${\text{h}}_{1}>\frac{1}{2}$ is impossible if ${\text{h}}_{2}>\frac{1}{2}$.

Lemma 2a: For weak selection coefficients and s_2_ ≤ s_1_ < 2s_2_, **T** can be subdivided into three regions: **T**_**1**_, **T**_**2**_, and **R**. **T**_**1**_ is a triangle similar to **T** with vertices at $\left(0,\frac{1}{2}\right)$, (0, 1) , and $\left(\frac{\alpha }{2},\frac{1}{2}\right).$ **T**_**2**_ is a right triangle with vertices at $\left(\frac{1}{2},\;0\right)$, (α, 0), and $\left(\frac{1}{2},1-\frac{1}{2\alpha }\right)$. **R** is formed by substracting **T**_**1**_ and **T**_**2**_ from **T**.

Proof:

**T**_**1**_ is formed by drawing a midline through **T** parallel to the h_1_ axis. By the midpoint theorem, the base of **T**_**1**_ has length one-half of the base of **T**, and is equal to $\frac{\alpha }{2}$.

**T**_**2**_ consists of the interval on h_1_ between $\frac{1}{2}$ and α, and forms an angle θ between the line ${\text{h}}_{1,\text{max}}=\alpha \left(1-{\text{h}}_{2}\right)$ and the h_1_-axis. The height of the right triangle so formed is given by $\left(\alpha -\frac{1}{2}\right)$ tan θ. As θ is also an angle of **T**, it is seen that tan $\theta =\frac{1}{\alpha }$. The height of **T**_**2**_ then simplifies to $1-\frac{1}{2\alpha }$, hence the vertex at $\left(\frac{1}{2},1-\frac{1}{2\alpha }\right)$.

Lemma 2b: For weak selection coefficients and ${\text{s}}_{1}\geq 2{\text{s}}_{2}$, **T** is subdivided into the two regions **T**_**1**_ and **R**, and **T**_**2**_ is entirely absent under these more asymmetric conditions. **T**_**1**_ is as described above and **R** is formed by subtracting **T**_**1**_ from **T**.

Proof: By assumption, ${\text{s}}_{1}\geq 2{\text{s}}_{2}$, and so $\alpha \leq \frac{1}{2}$. It follows that the base of **T** is less than or equal to $\frac{1}{2}$, and so **T** never crosses into the bottom right quadrant of the unit square.

Definition 1. P(x) is the *potential for polymorphism*, where P(x):= Area of x.

Definition 2. The SP conditions belonging in **T**_**1**_ and **T**_**2**_ are the “Cumulative Overdominance stability conditions”.

Definition 3. The SP conditions belonging in **R** are the “Reversal of Dominance stability conditions”.

Definition 4: The ratio $\text{U}\left(\alpha \right):=\frac{\text{P}\left({\text{T}}_{1}\right)+\text{P}\left({\text{T}}_{2}\right)}{\text{P}\left(\text{T}\right)}$ is the proportional contribution of the Cumulative Overdominance stability conditions to the overall potential for polymorphism P(**T**).

**Figure 2.**
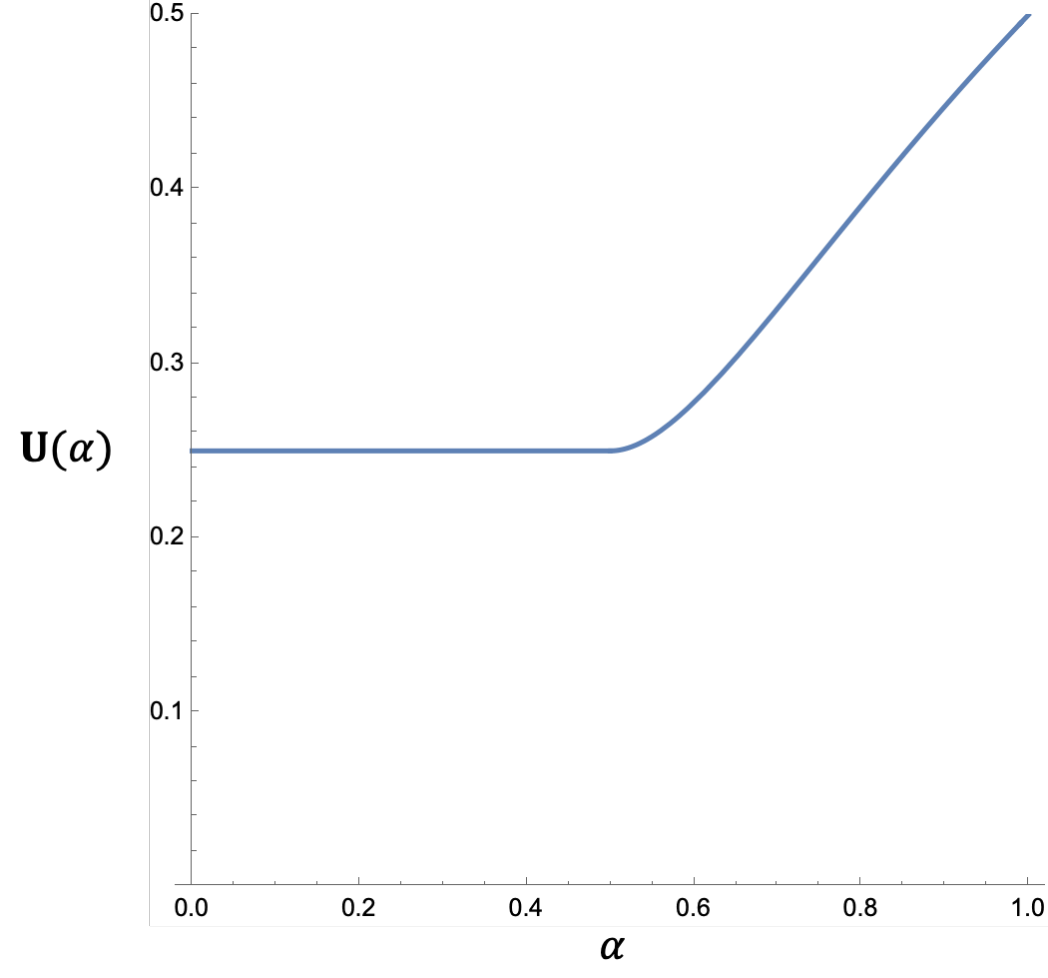
The proportional contribution of Cumulative Overdominance to the potential for polymorphism. The relative area of **T** residing in **T**_**1**_ and **T**_**2**_ is plotted for general values of the ratio of selection coefficients $\left(\alpha ={\text{s}}_{2}/{\text{s}}_{1}\right)$. The piecewise function ranges from 0.25 to 0.5.

Theorem: For weak selection coefficients, U(α) varies between 1/4 and 1/2, and is equal to the piecewise function (Figure 2):

(5) $$\text{U}\left(\alpha \right)=\{\begin{array}{ll} \frac{1}{4} & 0<\alpha \leq \frac{1}{2}\\ \frac{1}{4}\left(\frac{1}{\alpha^{2}}-\frac{4}{\alpha }+5\right) & \frac{1}{2}<\alpha \leq 1 \end{array}$$

Proof:

(6) $$P\left(\text{T}\right)=\text{ Area of }\text{T}=\frac{\left(\text{base of}\text{T}\right)\left(\text{height of}\text{T}\right)}{2}=\frac{\left(\alpha \right)\left(1\right)}{2}=\frac{\alpha }{2}$$

(7) $$P\left({\text{T}}_{1}\right)=\text{ Area of }{\text{T}}_{1}=\frac{\left(\frac{\alpha }{2}\right)\left(\frac{1}{2}\right)}{2}=\frac{\alpha }{8}$$

(8) $$\text{Case}\;1\left(0<\alpha \leq \frac{1}{2}\right):\;\text{P}\left({\text{T}}_{2}\right)=0,\text{ and therefore }\text{U}\left(\alpha \right)=\frac{\text{P}\left({\text{T}}_{1}\right)}{\text{P}\left(\text{T}\right)}=\frac{1}{4}$$

(9) $$\text{Case}\;2\left(\frac{1}{2}<\alpha \leq 1\right):\;\text{P}\left({\text{T}}_{2}\right)=\frac{\left(\alpha -\frac{1}{2}\right)\left(1-\frac{1}{2\alpha }\right)}{2},\text{and therefore }\text{U}\left(\alpha \right)=\frac{\text{P}\left({\text{T}}_{1}\right)+\text{P}\left({\text{T}}_{2}\right)}{\text{P}\left(\text{T}\right)}=\frac{\frac{\alpha }{8}+\frac{\left(\alpha -\frac{1}{2}\right)\left(1-\frac{1}{2\alpha }\right)}{2}}{\frac{\alpha }{2}}=\frac{1}{4}\left(\frac{1}{\alpha^{2}}-\frac{4}{\alpha }+5\right)$$

## Discussion

The proportion of parameter space that is stabilized owing to a constant direction of dominance was derived for the sex-independent seasonal selection model: 25% to 50% of all stability-conferring dominance schemes maintain a constant direction of dominance, with parity of selection coefficients tending towards the 50% figure and disparity tending towards the 25% figure.

### Generality and relation to other models of antagonistic pleiotropy

While the proofs presented here invoke weak selection, selection coefficients may range up to, say, 20% without significantly distorting the results. This owes to the fact that the curvature of the triangular stable regions remains negligible unless selection is rather intense (Fig 1c); deleterious dominance reversal contributes to the potential for polymorphism under these more extreme scenarios of fitness variation. Note that all results apply just as well to multivoltine populations that have an equal number of generations per season, the geometric mean fitness criterion being identical to that of the bivoltine model (Eqn. 1).

Supposing a single-locus model with a constant selection regime, antagonistic pleiotropy in which fitness components interact multiplicatively (e.g. an allele affecting both reproduction and viability, Hedrick 1999) is characterized by the same geometric mean fitness criterion as analyzed here, and so the conclusions carry over. For models of antagonistic pleiotropy in which the harmonic mean fitness of the heterozygotes must exceed that of the homozygotes to establish polymorphism (multiple-niche selection, Levene 1953), the conclusions apply without alteration under weak selection assumptions for the two-niche case; this owes to that fact that the identical h_1,max_ approximation is obtained upon ignoring products of selection coefficients. For antagonistic models in which the criterion is that the arithmetic fitness of the heterozygotes must exceed that of the homozygotes, the weak selection approximation obtained herein is general (that is, the h_1,max_) approximation is exact).

### Implications for modeling

As mentioned, the sizeable contribution of cumulative overdominance to stabilizing allelic oscillations is apparent when generalizing beyond the strict complement relation ${\text{h}}_{1}=1-{\text{h}}_{2}$. Indeed, parallel dominance provides, at worst, a quarter of the potential for polymorphism. If selection coefficients are not so disparate (α near 1), then the two mechanisms have roughly equal potentials. These conclusions merit a reconsideration of both (1) the definition of cumulative overdominance, and (2) the contribution of cumulative overdominance to stabilizing oscillations of allele frequencies.

Regarding the definitional issue, the cumulative overdominance mechanism has been described as “ incomplete dominance [reducing] selection against rare alleles in the environments they are unsuited to” (Bertram and Masel 2019, p. 884). Strict constancy of magnitude across seasons is unnecessary for dominance to operate along the spirit of this verbal description, as an overall constancy to direction (with changes in magnitude) is a form of incomplete dominance. Identifying cumulative overdominance as the intersection of the stable polymorphism conditions with the upper left and bottom right quadrants of the unit square (called “parallel dominance” in non-temporal models of antagonistic pleiotropy, Curtsinger et al. 1994), allows for all stability-conferring dominance schemes to be grouped into two classes (dominance reversal and cumulative overdominance/parallel dominance) with no regions left over (additivity in one or both seasons may be classified as cases of cumulative overdominance by convention, as Bertram and Masel 2019 do for the doubly additive case). Each of these classes is a subset of “geometric mean overdominance”, which is the condition given by Haldane and Jayakar (1963) (or Eqn 1, present article, as applies for two-season bivoltinism).

Terminology aside, the theorem of Eqn 5 is helpful in clarifying the role of the two genetic mechanisms in stabilizing seasonal polymorphism. Whereas several authors have concluded that constant dominance requires an exceedingly tight interval for small selection coefficients, the generalized schemes analyzed here lack this restrictive feature. The theorem makes clear that the argument of Bertram and Masel (2019), that cumulative overdominance preferentially stabilizes large-effect alleles due to the tight-interval effect, depends on the dominance coefficients hewing closely to the complement relation. Therefore, generalized dominance as a whole tends to suffer from the “weak-allele problem,” with exclusion of small-effect alleles occuring only for special restrictions on dominance. To the extent that mutations are “sampled” evenly from the whole of the dominance unit square, genetic mechanisms fare poorly as compared to ecological factors in preferentially stabilizing a system of many high amplitude oscillations.

The multilocus simulations of Wittmann et al. (2017) anticipated the general conclusion of the analytical results presented here. Namely, their Figure 1 0F reports a sizeable portion of dominance schemes that are off the complement line which are able to confer stability (in addition to a sizeable portion in the reversal of dominance region). They suggest the criterion $\left({\text{h}}_{1}+{\text{h}}_{2}\right)/2<0.5$ for predicting stable polymorphism, but this clearly fails under asymmetric selection coefficients. A satisfactory criterion was derived above: ${\text{h}}_{1}<\alpha \left(1-{\text{h}}_{2}\right)$, keeping in mind the “without loss of generality” assumption for labeling alleles.

In their study of the multiplicative model of antagonistic pleiotropy, Curtsinger et al. (1994) asked what percentage of selection parameter combinations are stabilized by the parallel dominance quadrants, finding approximately 25% of sampled selection pairs to be stabilized. This question is distinct from that posed in the present article, namely: for all dominance schemes in SP, what is the proportion of dominance schemes residing in the parallel dominance quadrants? Nevertheless, their numerical results clearly indicate the importance of non-reversing quadrants of the dominance unit square to the stability of polymorphism.

Van Dooren (2006) examined a single-locus high-dimensional phenotypic model in which allelic dominance is characterized by a vector of trait-specific values. He concluded that antagonistic pleiotropy and trait-specific dominance are generally required for stabilizing polymorphism. Importantly, he notes that the maximum possible ratio of stability-conferring parameter space with dominance reversal to that without dominance reversal “appears to be 3.” This is consistent with the 3:1 ratio of P(**R**) : P(**T**_**1**_) for $\alpha \leq \frac{1}{2}$ found here. Hence, the conclusions of a simple two-trait model (fitness in two seasons) seem to carry over not only to other classes of antagonistic pleiotropy, but to higher-dimensional phenotypes.

It remains to be discovered whether modifiers of the genetic system respond in a qualitatively different manner depending on which dominance model is operative.
