# Supplementary material for: Season-specific dominance broadly stabilizes polymorphism under symmetric and asymmetric multivoltinism
Source: bioRxiv. 2024 May 13:2023.11.20.567918. Originally published 2023 Nov 21. Preprint. [Version 2] doi: 10.1101/2023.11.20.567918 (PMC10690222; doi:10.1101/2023.11.20.567918)
Supplement: Supplement 1 [file media-1.pdf]

Supplemental Section 1 - The potential for polymorphism owing to dominance quadrants

Season-specific dominance broadly stabilizes polymorphism under symmetric and asymmetric multivoltinism

by Evgeny Brud

The absolute areas of the quadrant-specific subregions of P are derived by cases. These areas are each divided by the total area of P in order to obtain the relative areas of Box I.

Case i: $0 < \frac{s_1}{s_2} \leq \frac{1}{2}$

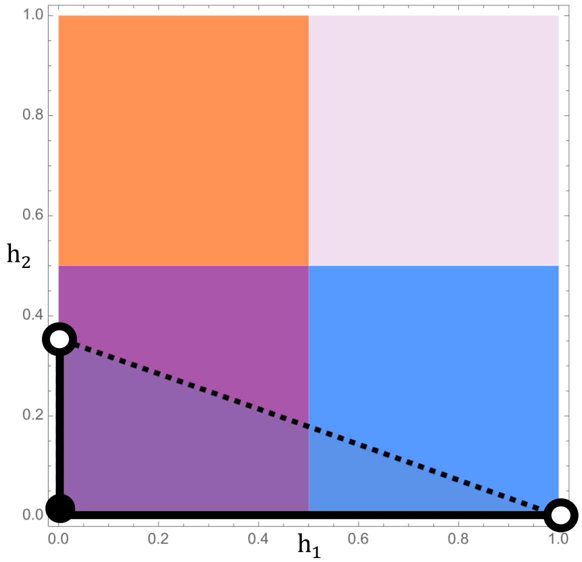

$$\text{Area(P)} = \int_0^1 \text{h}_{2,\max}\, d\text{h}_1$$

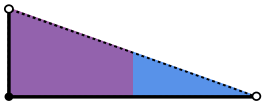

$$= \frac{(1-\text{s}_1)\log(1-\text{s}_1)+\text{s}_1}{\text{s}_1\text{s}_2}$$

$$\text{Area(P | a- dominance, case 1)} = \int_{1/2}^1 \text{h}_{2,\max}\, d\text{h}_1$$

$$= \frac{\left(\text{s}_1 + 2\text{s}_1\log\left(\frac{2-\text{s}_1}{2(1-\text{s}_1)}\right) - 4\coth^{-1}(3-2\text{s}_1) + \log(4)\right)}{2\text{s}_1\text{s}_2}$$

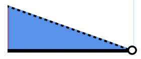

Area(P | beneficial reversal, case 1)

$$= \text{Area(P)} - \text{Area(P | a} - \text{ dominance, case 1)}$$

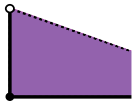

Case ii: $\frac{1}{2} < \frac{s_1}{s_2} \le \frac{2}{2+s_2}$

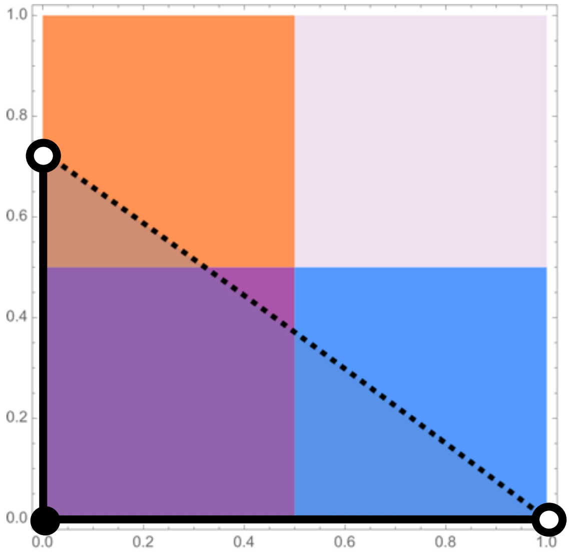

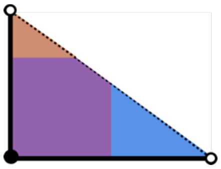

$$\text{Area(P)} = \int_0^1 h_{2,max}\, dh_1$$

$$= \frac{(1 - s_1)\log(1 - s_1) + s_1}{s_1 s_2}$$

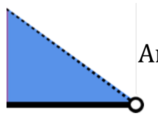

$$\text{Area(P | a- dominance, case 2)} = \int_{\frac{1}{2}}^{1} h_{2,max}\, dh_1$$

$$= \frac{s_1 + 2s_1 \log\left(\frac{2 - s_1}{2(1 - s_1)}\right) - 4\coth^{-1}(3 - 2s_1) + \log(4)}{2s_1 s_2}$$

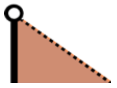

$$\text{Area(P | A- dominance,case 2)} = \int_{\frac{1}{2}}^{s_1/s_2} h_{1,max}\, dh_2$$

$$= \frac{\left(2s_1 - s_2 + 2\log(1 - s_1) - 2\log(2 - s_2) + 2s_1 \log\left(\frac{2 - s_2}{2(1 - s_1)}\right) + \log(4)\right)}{2s_1 s_2}$$

Area(P | beneficial reversal, case 2) =

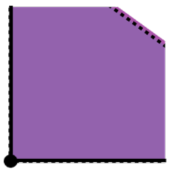

=

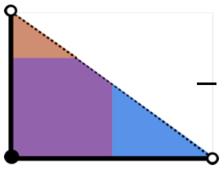

−

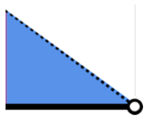

−

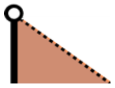

Case iii: $\frac{2}{2+s_2} < \frac{s_1}{s_2} \leq 1$

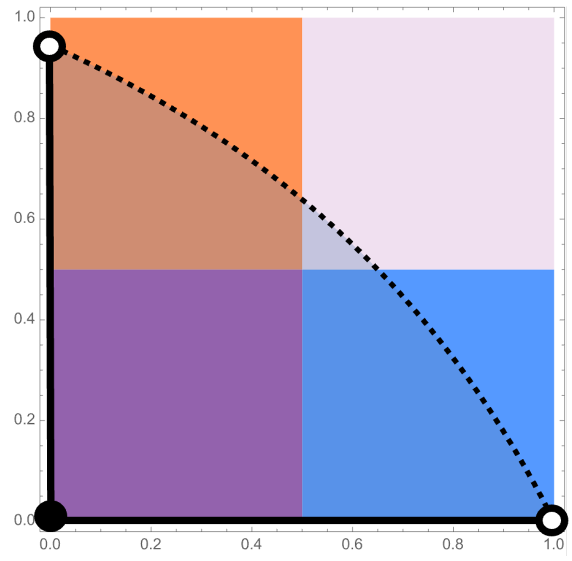

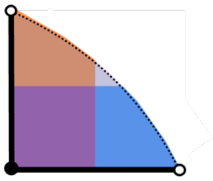

$$\text{Area(P)} = \int_0^1 h_{2,max}\, dh_1$$

$$= \frac{(1-s_2)\log(1-s_2)+s_2}{s_1 s_2}$$

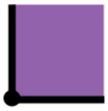

$$\text{Area(P | beneficial reversal, case 3)} = \frac{1}{4}$$

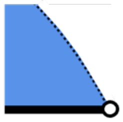 = 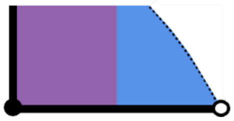 − 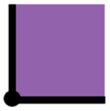

$$\text{Area(P | a} - \text{dominance, case 3)} = \int_0^{1/2} h_{1,max}\, dh_2 - \frac{1}{4}$$

$$= \frac{s_2 + 2\log(2-s_2) + 2s_1\log\left(\frac{2}{2-s_2}\right) - \log(4)}{2s_1 s_2} - \frac{1}{4}$$

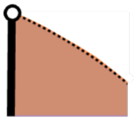 = 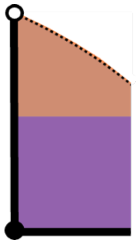 − 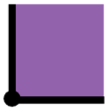

$$\text{Area(P | A} - \text{dominance, case 3)} = \int_0^{1/2} h_{2,max}\, dh_1 - \frac{1}{4}$$

$$= \frac{s_1 + 2\log(2-s_1) + 2s_1\log\left(\frac{2}{2-s_1}\right) - \log(4)}{2s_1 s_2} - \frac{1}{4}$$

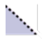 = 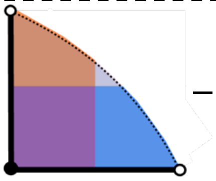 − 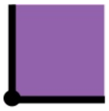 − 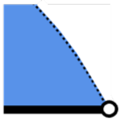 − 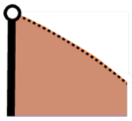

= Area(P | deleterious reversal, case 3)
