# Supplementary material for: Season-specific dominance broadly stabilizes polymorphism under symmetric and asymmetric multivoltinism
Source: bioRxiv. 2024 May 13:2023.11.20.567918. Originally published 2023 Nov 21. Preprint. [Version 2] doi: 10.1101/2023.11.20.567918 (PMC10690222; doi:10.1101/2023.11.20.567918)
Supplement: Supplement 2 [file media-2.pdf]

Supplemental Section 2 - Figures

Season-specific dominance broadly stabilizes polymorphism under symmetric and asymmetric multivoltinism

by Evgeny Brud

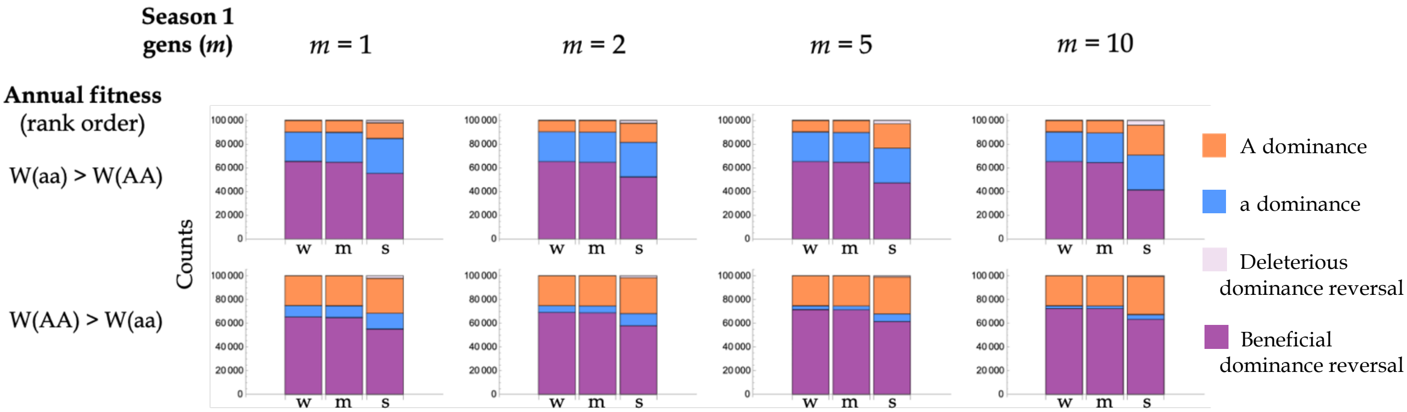

Figure S1 Numerical distribution of stabilizing dominance schemes under symmetric and asymmetric multivoltinism. Stacked bars indicate the counts for each category of season-specific dominance under weak (0-0.1%; w), moderate (0.1-10%; m), and strong (10-100%; s) ranges for the selection coefficients, assuming the region in Eqn 2 for the generation numbers (m, n) = (m, 1) and random uniform sampling of 10^5 points per parameter set. (Top row) Sampling assumes condition 7a for the rank order of homozygous annual fitnesses, i.e. W(aa)>W(AA). (Bottom row) Sampling assumes condition 7b, i.e. W(AA)>W(aa).

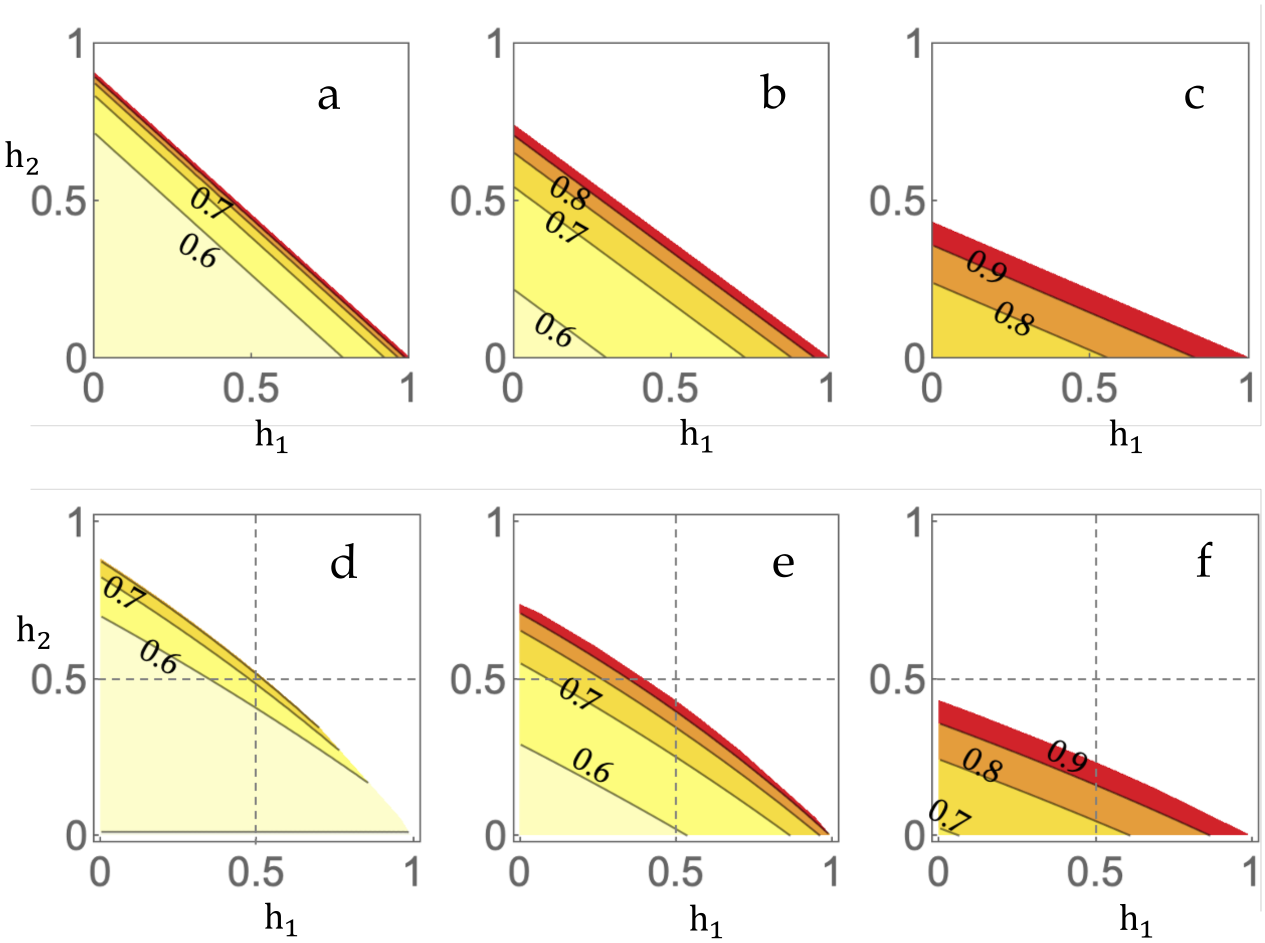

Figure S2 Equilibrium a-allele frequency at the start of season 1 in a bivoltine population. (a-c) The value of $\hat{q}_1$ is plotted for weak regimes of (s_1, s_2). (a) s_1 = 0.525%, s_2 = 0.475%, (b) s_1 = 0.575%, s_2 = 0.425% (c) s_1 = 0.7%, s_2 = 0.3%.(d-f) The numerical value of the a-allele frequency at generation 10^4 ($\hat{q}_{1_{\mathrm{num}}}$) is plotted under strong selection regimes. The biallelic model was iterated from q = 0.5 at gen 0. (d) s_1 = 32%, s_2 = 35%, (e) s_1 = 26%, s_2 = 35% (f) s_1 = 15%, s_2 = 35%.
